# HNRNPK/CLCN3 axis facilitates the progression of LUAD through CAF-tumor interaction

**DOI:** 10.7150/ijbs.76083

**Published:** 2022-10-17

**Authors:** Yixin Li, Yang Yang, Qian Ma, Huifang Cheng, Hui Wang, Chao Ma, Feng Li, Song Zhao, Xiangnan Li, Yu Qi, Zhuoyu Gu

**Affiliations:** 1 Department of Thoracic Surgery, The First Affiliated Hospital of Zhengzhou University, Zhengzhou University, Zhengzhou 450052, China.; 2 Department of Clinical Oncology, The First Affiliated Hospital of Zhengzhou University, Zhengzhou University, Zhengzhou 450052, China.; 3 Post-doctoral Station of Clinical Medicine, The First Affiliated Hospital of Zhengzhou University, Zhengzhou University, Zhengzhou 450052, China.

**Keywords:** LUAD, CLCN3, HNRNPK, Transcription, CAFs

## Abstract

**Background:** Chloride channel 3 (CLCN3) is regulated by transcription-coactivator, however, it is unclear which core transcription factor regulates CLCN3. The role of CLCN3 in lung adenocarcinoma (LUAD) is unexplored and the relationship between CLCN3 and tumor microenvironment is unknown.

**Methods:** A 5′-biotin-labeled promoter probe of CLCN3 was used to pull down the promoter-binding transcription factor. Further study was investigated using LUAD samples, cell lines, and xenograft mice models, and the mechanism was explored.

**Results:** CLCN3 was upregulated in human LUAD, and CLCN3 knockdown inhibited tumor proliferation and migration *in vitro*. Next, heterogeneous nuclear ribonucleoprotein K (HNRNPK) was first validated as a CLCN3 promoter-binding transcription factor. Mechanistically, HNRNPK knockdown suppressed the promoter activity of CLCN3, thus regulating CLCN3 expression at the transcriptional level, and the binding motif 'GCGAGG' and binding site '-538/-248 bp' were identified. Subsequently, the RNA-seq data illustrated that the primary functions of HNRNPK were similar to those of CLCN3. The results from *in vitro* and *in vivo* trials indicated that the expression and function of CLCN3 were regulated by HNRNPK. By isolating primary cancer-associated fibroblasts (CAFs) from human LUAD, we confirmed that decreased extracellular CLCN3 secretion induced by HNRNPK knockdown inhibited CAFs activation and TGF-β1 production, thus suppressing nuclear HNRNPK expression and LUAD progression in a feedback way. Furthermore, this phenomenon was rescued after the addition of TGF-β1, revealing that the HNRNPK/CLCN3 axis facilitated LUAD progression through intercellular interactions. Finally, we identified that CLCN3 and HNRNPK were upregulated and correlated with poor prognosis in LUAD patients.

**Conclusions:** HNRNPK/CLCN3 axis facilitates the progression of LUAD through CAF-tumor interaction.

## Introduction

Lung cancer (LC) is an extremely aggressive malignancy and a primary cause of global cancer-related mortality 1. LC can be classified as either non-small cell lung cancer (NSCLC) or small cell lung cancer, though NSCLC consists of the majority of LC patients and lung adenocarcinoma (LUAD) is the most prevalent pathological subtype 2. Despite major improvements in the diagnosis and clinical treatment of LUAD, patients' 5-year survival rate is dismal, due to difficulty in early detection, high metastasis rates, resistance to radiotherapy and chemotherapy, and lack of systematic treatment 3. Therefore, achieving detailed molecular mechanisms of LUAD development and identifying new molecular markers are essential for improved diagnosis and treatment of LUAD.

Chloride channels are the most key anion transporters in plasma membranes by mediating transmembrane transport of biomolecules 4, 5, however, the functions and mechanisms of action of chloride channels in LUAD are not fully understood. Chloride channel 3 (CLCN3), one of the members of the chloride channel family that are voltage-gated, primarily regulates the homeostasis state of intercellular ions and the acidification of cellular compartments 6, 7. CLCN3 has been shown to function in cell proliferation, migration, and resistance to pharmacological treatment in glioma, ovarian cancer, and stomach adenocarcinoma cells 8-10. Therefore, CLCN3 may function critically in the initiation and development of cancer. Our previous study indicated that CLCN3 can be regulated by the transcription-coactivator XRCC5 11, but it is still unclear which core transcription factor regulates CLCN3 and how it functions in LUAD.

Heterogeneous nuclear ribonucleoproteins (hnRNPs) represent a family of RNA-binding proteins that participate in pre-mRNA processing, mature mRNA transport, mRNA transcription, and translation 12. hnRNPs are associated with cell proliferation, cell invasion, oxidative stress, and metabolic reprogramming 13-15, yet the function of hnRNPs in respiratory tumors, specifically LUAD, has not been completely clarified. One family member, HNRNPK, regulates a multitude of cellular functions, including DNA/RNA binding, transcription, and nucleocytoplasmic shuttling 13. In various types of malignancies, HNRNPK has been characterized as an oncogene and predicts poor survival, however, it was recently shown to function as a tumor suppressor in hematologic malignancies 13. Here, HNRNPK is identified as a CLCN3 promoter-binding transcription factor. However, the relationship between CLCN3 and HNRNPK has not been reported yet. It is unclear whether HNRNPK/CLCN3 axis is essential for LUAD progression, and its prognostic significance is unknown.

The tumor microenvironment (TME) contains tumor, stromal, and immune cells 14. The majority of the cells that make up the stroma are known as cancer-associated fibroblasts (CAFs). CAFs are similar to myofibroblasts and are known to drive cancer progression in the TME 15. Nonetheless, there is a paucity of information about the link between CLCN3 and CAFs. In this study, we demonstrate that decreased extracellular CLCN3 secretion inhibited CAFs activation, thus suppressing nuclear HNRNPK expression and LUAD progression in a feedback way. Additionally, both CLCN3 and HNRNPK were upregulated and linked to unfavorable prognosis in LUAD patients. This study suggested that HNRNPK/CLCN3 axis facilitated the LUAD progression through tumor cell interactions with CAFs. The aim was to uncover novel molecular biological mechanisms of LUAD progression and provide novel therapeutic targets.

## Methods

### Patients and samples

Beginning from May 2020 to April 2021, fresh tumor tissues and ANT were acquired from 16 LUAD patients at the First Affiliated Hospital of Zhengzhou University. Approval for this research was granted by the ethics committee of the First Affiliated Hospital of Zhengzhou University (2022-KY-0109-004), and all patients who participated in the research project gave their written informed consent. In addition, a human LUAD tissue microarray consisting of 30 cases was purchased from Superchip (Shanghai, China).

### Culture of cells and establishment of stable cell lines

After acquiring the human LUAD cell lines (H1299, A549, and PC-9), authentication was done using STR profiling from Shanghai GenePharma Co. (Shanghai, China). Additionally, Prof. Wuguo Deng (Sun Yat-sen University Cancer Center) supplied the human bronchial epithelial cell line (HBE). All cells were grown in RPMI-1640 media that had been supplemented with 10% fetal bovine serum (FBS), and the mixture was placed in an incubator with a humidified environment that contained 5% carbon dioxide. Lentivirus vectors were procured from Shanghai GenePharma Co. (Shanghai, China), including CLCN3 knockdown (5´-GCAGGCATTGGAGTATATTCA-3´), HNRNPK knockdown (shHNR-1: 5´-3´AGATTTGGCTGGATCTATT; shHNR-2: 5´-CGTTATTGTTGGTGGTTTA-3´), and HNRNPK overexpression vectors. Through the use of selection containing 1 μg/mL puromycin for four weeks, stable cell lines were generated. Within an hour, CAFs and normal fibroblasts (NFs) were produced from fresh LUAD tissues as well as nearby normal tissues (which were at a minimum of 5 cm distant from the site of the malignant tissue). Primary cell isolation was performed as described in our previously published articles, and the cells were passaged for approximately 5-7 populations 16. CAFs and NFs were cultured in DMEM/F12 medium containing 15% FBS.

### Streptavidin-agarose-mediated DNA pull-down assay

Ruibiotech Co. (Beijing, China) developed a biotin-labeled, double-stranded oligonucleotide probe that corresponds to the -248 to -226 segment of the CLCN3 promoter sequence. To determine protein binding to the promoter, we performed a DNA pull-down assay as follows. Specifically, A total of 1 mg of nuclear protein extract, 10 μg of the probe, and 100 μL of streptavidin-agarose beads (Sigma, St Louis, MO) were mixed together and subjected to incubation. This was followed by the centrifugation of the mixture at 800×g before resuspending it in 30 μL of loading buffer and boiling it for 5 minutes at 100 °C. Using SDS-PAGE, we isolated the bound proteins from the sample, and the gels were either silver stained or processed for western blotting (WB).

### Silver staining and mass spectroscopy (MS)

After placing and covering the protein gel in a solution consisting of 50% ethanol, 40% water, and 10% acetic acid, a shaker was used to incubate the mixture at ambient temperature throughout the night. After that, the gel was silver stained and thereafter evaluated via liquid chromatography-tandem mass spectrometry (LC-MS/MS) from Wininnovate Bio-Tech Co., Ltd. (Shenzhen, China).

### Chromatin immunoprecipitation (ChIP) and dual-luciferase reporter assay

A ChIP kit was used as per the guidelines stipulated by the manufacturer (#9002S, Cell Signaling Technology, Danvers, MA). Once the samples had been cooled on ice, the DNA was separated by sonication into sizes ranging from 100 to 1000 base pairs (bp). Following the addition of antibodies to a final dilution of 1:50, the solution was incubated at 4 °C throughout the night, after which it was re-incubated with protein G agarose beads at the same temperature overnight. After a series of rinses, the DNA-protein mixtures that had been bound were eventually eluted, and the cross-linking reaction was reversed. This was followed by the purification of the DNA fragments, and qRT-PCR was utilized to determine the level of CLCN3 based on the primers for the CLCN3 promoter: 5´-GCTTTTGGTGCTGGTTTAT-3´ (forward), 5´-CTAATCGCTAATGACAGGC-3´ (reverse). The input samples represented the positive control, whereas normal rabbit IgG was employed as the negative control in this experiment. The precipitated chromatin DNA was collected and analyzed via ChIP-seq aligned to the human genome (Seqhealth Co., Wuhan, China). We submitted the ChIP-seq data to the NCBI Gene Expression Omnibus (GEO) (accession number GSE197777). For the dual-luciferase reporter assay, the primer pairs for the truncated promoter regions of CLCN3 and the protocol are described in our previous study 11.

### RNA extraction and quantitative RT-PCR

Isolated total RNA from cells were thereafter reverse-transcribed into cDNA with the help of the ReverTra Ace qPCR RT Master Mix kit (Toyobo, Japan) and primers for CLCN3 (cat#HQP001983), HNRNPK (cat#HQP008957), and GAPDH (cat#HQP006940) (GeneCopoeia, Inc., USA). Lastly, in a CFX96 real-time PCR system (Bio-Rad), quantitative real-time PCR was conducted utilizing the SYBR® Green Real-time PCR Master Mix (Toyobo). The fold changes were determined using the 2^-ΔΔCT^ method, and they were used to compute relative RNA levels.

### Immunoprecipitation (IP) and western blotting (WB)

On a revolving wheel, nucleoprotein extracts were produced and then incubated for 24 hours using antibodies against HNRNPK at a 1:50 dilution or with IgG. After that, the sepharose-conjugated protein-A/G beads were introduced, and the mixture was then incubated on a revolving disc at a temperature of 4 °C for an additional twenty-four hours. Following a thorough washing using ice-cold PBS and boiling of the beads, the precipitate proteins were subjected to SDS-PAGE for separation before being deposited onto PVDF membranes. After blocking the membranes using nonfat milk at a concentration of 5% for two hours at ambient temperature, the membranes were then treated with primary antibodies at a dilution of 1:500 and secondary antibodies, and enhanced chemiluminescence was used to identify protein bands. The following primary antibodies were utilized in this study: CLCN3 (Novus, #NBP1-91790, 1:500), IgG (CST, #2729, 1:1000), HNRNPK (Proteintech, #11426-1-AP, 1:1000), AKT (Proteintech, #10176-2-AP, 1:1000), p-AKT (CST, #4060, 1:1000), α-SMA (Abclonal, #A17910, 1:1000), COL1A1 (Abclonal, #A16891, 1:1000), FAP (Abclonal, #A6349, 1:1000), β-Actin (Proteintech, #20536-1-AP, 1:2000) and Histone H3 (Abclonal, #A2348, 1:1000), GAPDH (Abclonal, #A19056, 1:1000).

### Cell proliferation, MTS, and colony formation assays

The IncuCyte live-cell imaging system (Essen BioScience) was utilized initially for the purpose of measuring the rate of cell proliferation. Phase microscopy (NiKon, #MRH00101) was used to capture cell proliferation images and curves after the cells had been grown in 96-well plates and put in the IncuCyte system. This was followed by analysis using the IncuCyte analysis software. To complete the MTS assay, the cells were plated at a density of 5,000 cells/well. Following a seeding duration of 1-3 days, the cells were subjected to 40 minutes of incubation with MTS (Promega, Madison, WI) for 40 minutes at each of those time points. Additionally, with the aid of a microplate reader, the optical density (OD) was measured. To facilitate the formation of colonies, the cells were planted in 6-well plates at 500 cells/well. After incubating the cells for two weeks, 4% paraformaldehyde was utilized to fix them before staining them with crystal violet. Images of the colonies were captured and processed with Image-Pro Plus 6.0 (Media Cybernetics, MD, USA) to facilitate the counting of the total colonies.

### Scratch wound healing and transwell assays

The cells were grown in 6-well plates until they reached confluence, and afterward, a 10-μL pipette tip was used to scrape through the cell layer that was present in each well. After taking measurements of the gap widths at 0 hours (width 1(w1)) and 36 hours (width 2(w2)), the relative rate of migration was computed as follows: (w1 - w2)/w1 × 100%. To execute a transwell migration assay, Boyden chambers that included 24-well transwell plates were used (BD, USA). The top chambers that had been covered with Matrigel were then introduced with homogenous single-cell suspensions. Once 36 h elapsed, crystal violet was used to stain the cells that had invaded through the membrane in the bottom chambers. The number of cells in each of the five random fields was counted.

### RNA-seq

Total RNA was extracted and assessed for quality control. On an Illumina HiSeq platform, RNA-seq was carried out using the DGE method, and paired-end reads of 50 base pairs were produced by BGI Co. (Shenzhen, China). The data obtained by deep sequencing were uploaded to the NCBI Gene GEO database with the accession number GSE197845 (H1299 cells transfected with control or CLCN3 shRNA), GSE197946 (H1299 cells transfected with control or HNRNPK shRNA), and GSE197540 (CAFs stimulated with control or HNRNPK knockdown supernatants).

### *In vivo* tumor model

To establish xenograft models, H1299 cells were diluted at about 2 × 10^6^ in 100 μL of PBS and administered to the female BALB/c nude mice via subcutaneous injection. The volume of the tumor was measured every 4 days, and its volume was computed as follows: volume = (width^2^ × length) / 2. Once 25 days had passed, the tumor xenografts were excised, after which they were weighed and subjected to IHC staining. To establish lung metastasis models, through the tail vein of female BALB/c nude mice, an injection of H1299 cells containing 1.5 × 10^6^ cells in 100 μL of PBS was performed. Both bioluminescence imaging and histological analysis were used to investigate lung metastasis. Using IVIS Spectrum (Perkin Elmer, USA) for quantitative luminescence analyses, the normalized fluorescence intensities of lung tissues were measured and directly represented as the average radiance (photon/ second/ cm^2^/ steradian, or p/s/cm^2^/sr). The areas of metastatic foci in histopathological images were digitalized and calculated by using ImageJ software (NIH, MD, USA) for metastatic intensity analyses and quantified as the lung metastasis area (mm^2^). Every experiment involving animals was undertaken in compliance with the institutional ethical standards for animal studies that had been adopted by Zhengzhou University.

### Immunohistochemical (IHC) and immunofluorescence (IF)

Following overnight incubation, tissue slices were subjected to incubation with primary antibodies diluted at a ratio of 1:100. The tissue slices were washed and treated with specific secondary antibodies before staining with diaminobenzidine (DAB) or 4,6-diamidino-2-phenylindole (DAPI). Next, the tissue slides were scored independently by two investigators. For IHC or IF scoring, A score between 0 and 300 was obtained by multiplying the proportion of tumor cells that were stained (ranging from 0% to 100%) by the intensity (ranging from 1 to 3). Kaplan-Meier survival curves were used to assess the prognosis. After seeding the cells onto coverslips in a 6-well plate, they were then fixed with paraformaldehyde, followed by permeabilization with 0.5% Triton-X, and blocking with bovine serum albumin (BSA). The cells were thereafter subjected to overnight incubation with primary antibodies diluted at a ratio of 1:50. The following day, the cells were rinsed, then treated with secondary antibodies that had been fluorescently tagged, and finally stained with DAPI. A confocal microscope (Olympus, Japan) was utilized to obtain all images.

### GEPIA, cBioPortal, and Human Protein Atlas (HPA) datasets

The GEPIA dataset of TCGA gene expression (http://gepia.cancer-pku.cn) was used for evaluating the RNA-seq data of expression in tumor and normal tissues and the correlation between CLCN3 and HNRNPK in LUAD tissues was calculated. The cBioPortal for Cancer Genomics datasets (www.cbioportal.org) is a publicly accessible website for interactive exploration of data on RNA expression, DNA methylation, protein levels, and clinical data. A total of 181 patients diagnosed with LUAD (OncoSG, Nat Genet 2020) were used to visualize and analyze the correlation between CLCN3 and HNRNPK. The methods used for analysis were based on the online instructions provided by the website. The survival information of 500 clinical LUAD patients, including 270 females and 230 males, was obtained from the HPA database (https://www.proteinatlas.org/). The overall survival (OS) of patients with LUAD was evaluated following the online instructions.

### Statistical analysis

SPSS (version 17.0), a statistical software tool, was utilized to execute the necessary analyses of statistical data. The presentation of all of the data was in the form of mean ± SD. Either a two-tailed Student t-test or a variance analysis was employed to determine whether or not the differences were significant. The Spearman rank correlation analysis was performed to evaluate the correlation, and the Kaplan-Meier analysis was utilized to evaluate the OS curves. When *P* was < 0.05, statistical significance was attained.

## Results

### CLCN3 was upregulated in human LUAD and facilitated tumor proliferation and migration

**Figure 1:**
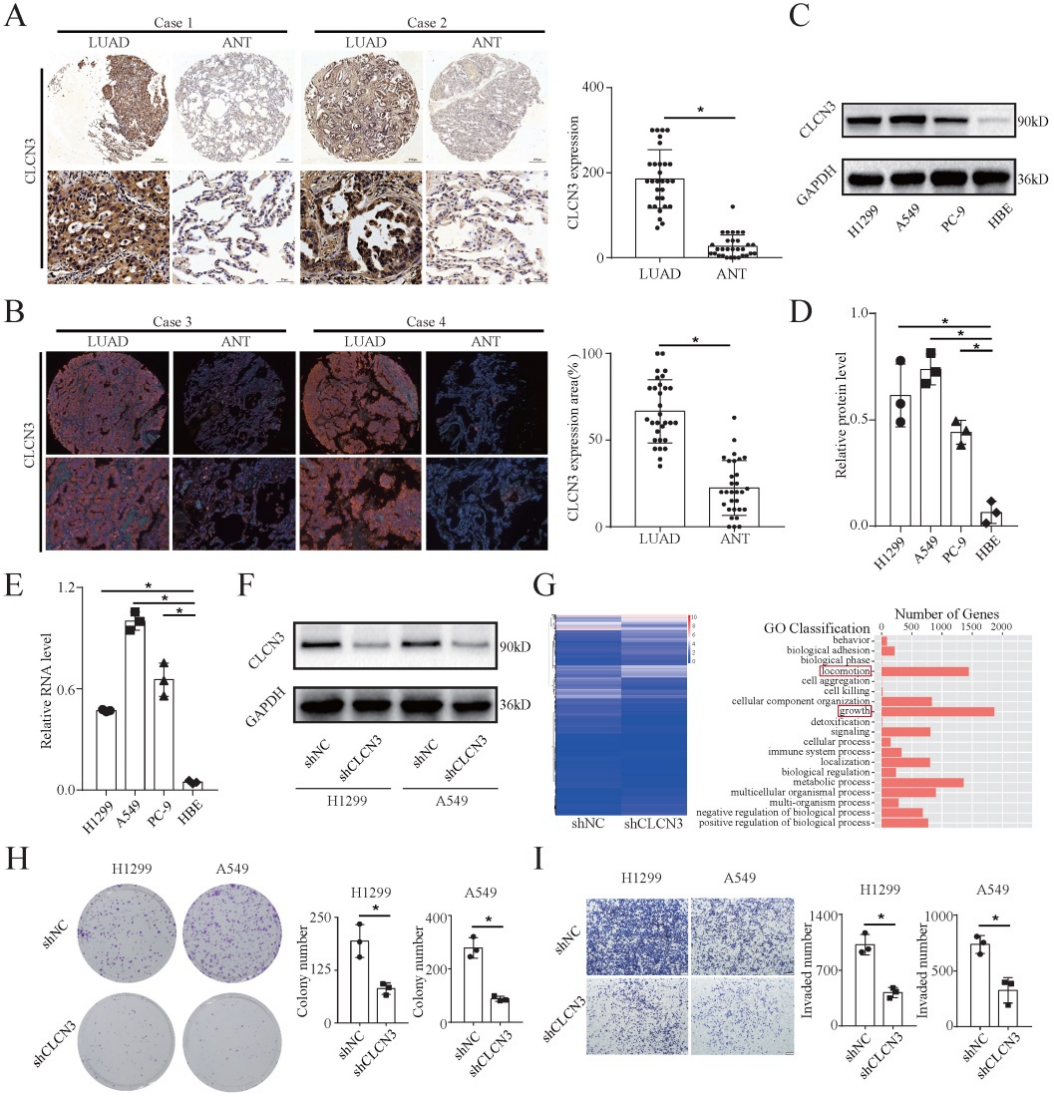
** CLCN3 was upregulated in human LUAD and facilitated tumor proliferation and migration. (a, b)** Through IHC and IF analysis, the expression of CLCN3 was examined in a tissue microarray of 30 paraffin-embedded LUAD tissues and adjacent normal tissues (ANT) (*n* = 30). **(c, d)** In human LUAD cell lines as well as in human bronchial epithelial cell lines, the basic protein expression of CLCN3 was measured (*n* = 3). **(e)** The basic RNA level of CLCN3 was detected in human LUAD cell lines and a human bronchial epithelial cell line (*n* = 3). **(f)** The protein expression of CLCN3 was inhibited after CLCN3 knockdown in H1299 and A549 cells. **(g)** RNA-seq was constructed after CLCN3 knockdown in H1299 cells. Locomotion and growth were significantly enriched as illustrated by GO analysis. **(h)** Knockdown of CLCN3 suppressed the clonogenicity of H1299 and A549 cells (*n* = 3). **(i)** CLCN3 knockdown suppressed the invasion of H1299 and A549 cells (*n* = 3). *P < 0.05.

We examined CLCN3 expression in a tissue microarray consisting of 30 LUAD tissues and adjacent normal tissues (ANT) using IHC and IF analysis. Notably, the expression level of CLCN3 was shown to be elevated in LUAD tissues and was found to be primarily localized within the cytoplasm (**Fig. 1a, 1b**). In the HBE and human LUAD cell lines (H1299, A549, and PC-9), the basic expression level of CLCN3 was elevated in LUAD cells in contrast with normal cells, as detected by WB (**Fig. 1c, 1d, S1a**). The RNA level for CLCN3 was comparable to the protein expression level of CLCN3 in tissues and cell lines (**Fig. 1e**). Collectively, these findings indicated that CLCN3 was upregulated in LUAD. To examine the functional role of CLCN3 in LUAD, we constructed H1299 and A549 cells with stable knockdown of CLCN3 (shCLCN3) and control cells (shNC) (**Fig. 1f, S1b**). By RNA sequencing (RNA-seq) analysis, a total of 3006 differentially expressed genes (DEGs) were identified in H1299 cells, including 1681 upregulated and 1325 downregulated genes. KEGG pathway analysis identified enrichment of DEGs in many signal transduction molecules and networks. In particular, locomotion and growth were significantly enriched as illustrated by GO analysis (**Fig. 1g, S1c**). Thus, we further examined the role of CLCN3 in tumor proliferation and migration *in vitro*. CLCN3 knockdown decreased the clone-forming number of LUAD cells (**Fig. 1h**), and markedly inhibited the cell proliferation within 24 h, as detected in clone formation and MTS assays, respectively (**Fig. S1d, S1e**). Similarly, transwell and scratch wound-healing assays illustrated that CLCN3 knockdown dramatically reduced cell invasion and migration in contrast with control cells (**Fig. 1i, S1f**). Thus, CLCN3 facilitated tumor proliferation and migration, exerting an oncogenic role in human LUAD.

### HNRNPK was identified and validated as a CLCN3 promoter-binding transcription factor

**Figure 2:**
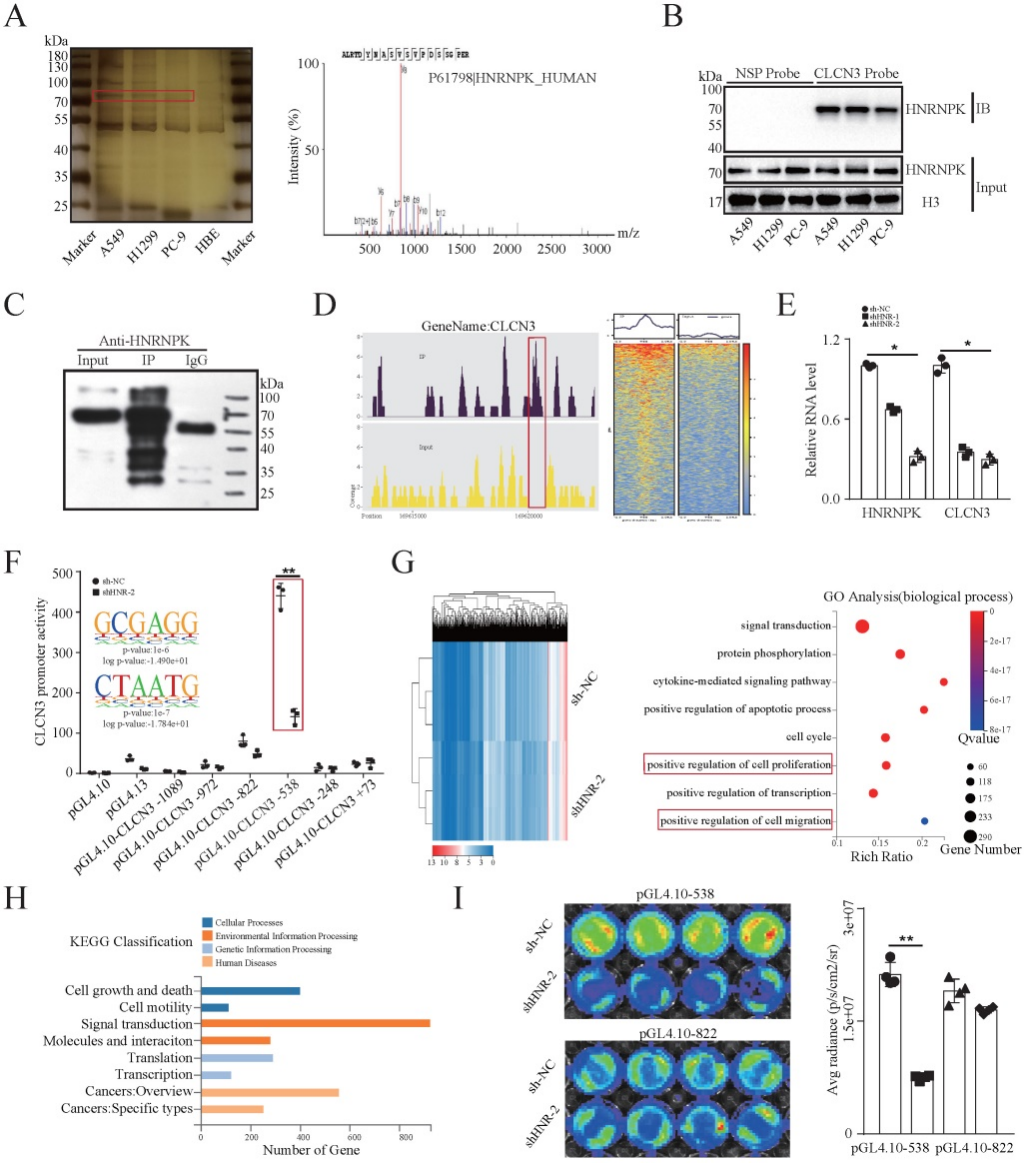
** HNRNPK was identified and validated as a CLCN3 promoter-binding transcription factor. (a)** To identify putative transcription factors that bind to the CLCN3 promoter, nuclear protein extracts were subjected to incubation with a biotin-labeled DNA probe that included the promoter region. Following the SDS-PAGE separation and silver staining, a protein band with differential expression was identified after which it was excised and subjected to mass spectrometry (MS) analysis. **(b)** By employing either a synthesized probe or a nonspecific probe (NSP) in the nuclear protein/DNA complex, western blot analysis detected binding between HNRNPK and the CLCN3 promoter. **(c)** ChIP assays were performed and an HNRNPK antibody was used to immunoprecipitate the fragmented DNA in H1299 cells. The HNRNPK protein band was substantially enriched in the IP group in contrast to the IgG control. **(d)** The ChIP-seq data showed that the recruitment of HNRNPK was mostly enriched in the upstream and downstream TSS of the CLCN3 promoter region (NM_001829, chr4: 169620033-169620797, region -538/+226 bp). **(e)** The CLCN3 RNA level was inhibited following HNRNPK knockdown (*n* = 3). **(f)** HNRNPK knockdown disrupted the promoter activities of the pGL4.10-CLCN3-538 reporter plasmid (region -538/+226 bp) in H1299 cells, and the binding motifs 'GCGAGG/CTAATG' from ChIP-seq data were enriched in this region (*n* = 3). **(g)** GO analysis revealed that cell proliferation and migration were the biological functions of HNRNPK. **(h)** KEGG pathway analysis illustrated that cell growth and death, cell motility, transcription, translation, and other signaling pathways were mainly enriched after HNRNPK knockdown. **(i)** The luciferase activity was considerably reduced in HNRNPK knockdown cells transfected with a pGL4.10-CLCN3-538 plasmid (n = 3). *P < 0.05, **P < 0.01.

To evaluate the transcriptional regulatory mechanism underlying CLCN3 expression in LUAD, a 5′biotin-labeled DNA probe corresponding to CLCN3's promoter region between -248 and +226 was incubated with nuclear protein extracts to identify possible transcription factors binding to the CLCN3 promoter. Following SDS-PAGE separation and silver staining, we observed a differentially expressed protein (at a molecular weight of almost 75 kDa, red rectangle), which was considerably enriched in LUAD cells in contrast with the normal cells (**Fig. 2a, left**). After that, an excised section of the protein band was examined using mass spectrometry (MS). As a potential protein that is bound to the CLCN3 promoter, the transcription factor HNRNPK was found to have the optimal peptide-spectrum sequence, which was ALRTDYNASVSVPDSSGPER (**Fig. 2a, right**). We identified the nuclear protein/DNA complex in LUAD cells utilizing a manufactured specific DNA probe or a nonspecific probe (NSP) and verified the binding using western blot to further define the binding that occurs between HNRNPK and the CLCN3 promoter (**Fig. 2b**). In addition, an assay was performed using a specific antibody against HNRNPK to immunoprecipitate DNA fragments in H1299 cells. First, we identified that the HNRNPK protein band was substantially enriched in the IP group in contrast with the IgG (negative control) antibody (**Fig. 2c**). Second, DNA fragments were extracted, and the ChIP-seq DNA libraries were successfully constructed. The standard pie chart for peak annotation revealed that over half of the peaks were located within the enhancer regions (intronic and intergenic regions), and 6.71% of the peaks were distributed in the promoter regions (**Fig. S2a**). In addition, the typical transcription start site (TSS) enrichment plot showed that compared with the input fragments, IP fragments were mainly enriched at TSS (**Fig. S2b**). Importantly, the ChIP-seq data showed that the recruitment of HNRNPK was mostly enriched in the upstream and downstream regions of TSS of the CLCN3 promoter region (NM_001829, chr4: 169620033-169620797, region: -538/+226 bp) (**Fig. 2d**). Finally, the ChIP-qPCR data showed that following HNRNPK knockdown, the expression of CLCN3 RNA was suppressed, which provided evidence that the expression of CLCN3 is mediated by HNRNPK at the transcriptional level (**Fig. 2e**).

Previously, a series of truncated CLCN3 gene promoter dual-fluorescence reporter plasmids were constructed 11. The dual-luciferase reporter assay illustrated that silencing HNRNPK in H1299 cells compromised the promoter activity of the pGL4.10-CLCN3-538 reporter plasmid (region, -538/+226bp), in which the binding motifs 'GCGAGG/CTAATG' from the ChIP-seq data were enriched (**Fig. 2f**). In further exploration of the function of HNRNPK in LUAD, Gene Ontology (GO) analysis identified the biological functions of HNRNPK to be positive regulation of cell proliferation and migration. Additionally, signal transduction, protein phosphorylation, apoptotic process, cell cycle and transcription were enriched and associated with HNRNPK (**Fig. 2g**). Gene set enrichment analysis (GSEA) demonstrated that the growth and locomotion significantly correlated with HNRNPK knockdown (**Fig. S2d, S2e**). KEGG pathway analysis illustrated a substantial enrichment of DEGs mainly in cell growth and death, cell motility, transcription, translation, and other signaling pathways (**Fig. 2h**). Next, a considerable reduction in luciferase activity was observed in HNRNPK knockdown cells transfected with the pGL4.10-CLCN3-538 plasmid, though not with the pGL4.10-CLCN3-822 plasmid (**Fig. 2i**). Altogether, these findings highlighted that HNRNPK was the CLCN3 promoter-binding transcription factor, and the primary functions of HNRNPK were like those of CLCN3.

### HNRNPK knockdown inhibited the expression and function of CLCN3 *in vitro*

**Figure 3:**
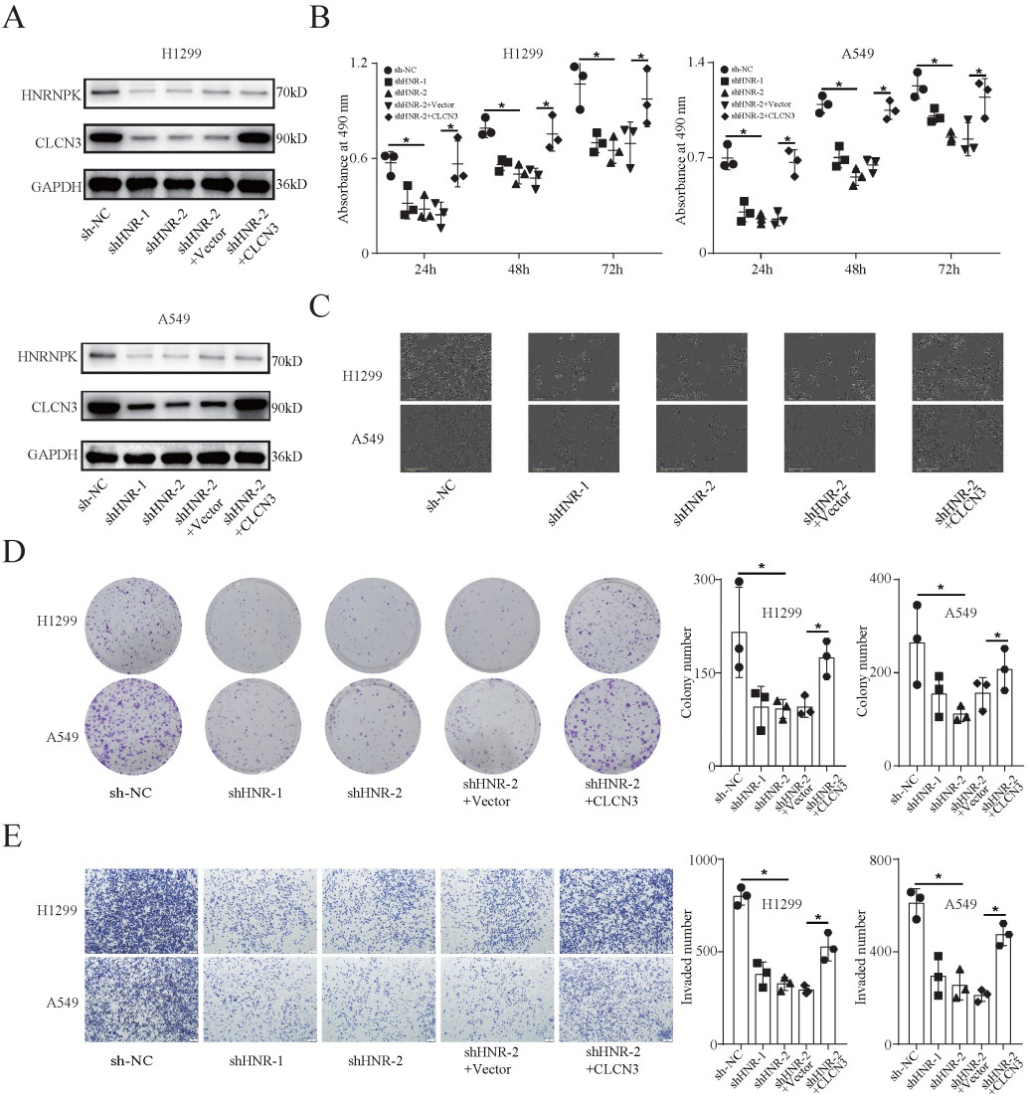
** HNRNPK knockdown inhibited the expression and function of CLCN3 *in vitro*. (a)** The expression of CLCN3 was inhibited after HNRNPK knockdown but this inhibition was abrogated by CLCN3 upregulation. **(b)** Following HNRNPK knockdown, there was a decrease in the proliferation of LUAD cells, however, this decrease was restored by the upregulation of CLCN3 (*n* = 3). **(c)** The representative cell images showed that HNRNPK knockdown inhibited the cell proliferation at 24 h and was rescued by CLCN3 overexpression. **(d)** Cell clonogenicity was alleviated as a result of HNRNPK knockdown, and this reduction was restored by the upregulation of CLCN3 (*n* = 3). **(e)** The cell invasion was suppressed following HNRNPK knockdown, and this inhibition was rescued by CLCN3 upregulation (*n* = 3). *P < 0.05.

To study the functional regulation of CLCN3, we established stable HNRNPK-knockdown cell lines (H1299 and A549) and corresponding rescue models by transfecting cells with the CLCN3 overexpression lentivirus. Here, we chose shHNR-2 to establish rescue models, as targeting this fragment significantly attenuated the promoter activity of CLCN3 in Figure 2f. WB analysis confirmed that the CLCN3 expression was suppressed following HNRNPK knockdown, and the inhibitory effect was reversed by CLCN3 overexpression (**Fig. 3a**). Moreover, following HNRNPK knockdown, LUAD cell proliferation was reduced, however, this effect could be restored by CLCN3 overexpression (**Fig. 3b, 3c**). Also, an IncuCyte-based analysis was employed to conduct reliable monitoring of cell proliferation and it was revealed that cell proliferation was attenuated following HNRNPK knockdown, but that this reduction could be restored by CLCN3 overexpression (**Fig. S3a, S3b**). In addition, HNRNPK knockdown decreased cell clonogenicity, although CLCN3 overexpression reversed this attenuation effect (**Fig. 3d**). Transwell and scratch wound healing assays also illustrated that cell invasion and migration were suppressed following HNRNPK knockdown, and the inhibitory action was reversed by CLCN3 overexpression (**Fig. 3e, S3c, S3d**). Overall, these data demonstrated that the expression and function of CLCN3 were suppressed following HNRNPK silencing *in vitro*.

### HNRNPK overexpression promoted the expression and function of CLCN3 *in vitro*

To additionally examine if CLCN3 is the target of HNRNPK, we generated a stable HNRNPK-overexpression cell line (PC-9) and corresponding rescue model by transfecting cells with the CLCN3 knockdown lentivirus. WB analysis demonstrated that expression of CLCN3 increased following HNRNPK overexpression, and this increase was reversed by CLCN3 knockdown (**Fig. S4a**). The MTS assay, clone formation assay, IncuCyte data, and cell proliferation images revealed that HNRNPK overexpression enhanced the proliferation as well as the clonogenicity of LUAD cells, and CLCN3 knockdown reversed these promoting effects (**Fig. S4b, S4c, S3e**). In addition, transwell and scratch wound healing assays highlighted that cell invasion and migration were enhanced following HNRNPK overexpression, and these promoting impacts were abrogated by CLCN3 knockdown (**Fig. S4d, S4e**). Collectively, these data illustrated that the expression and function of CLCN3 were enhanced following HNRNPK upregulation* in vitro*.

### HNRNPK/CLCN3 axis facilitated LUAD progression through the interaction between tumor cells and CAFs

**Figure 4:**
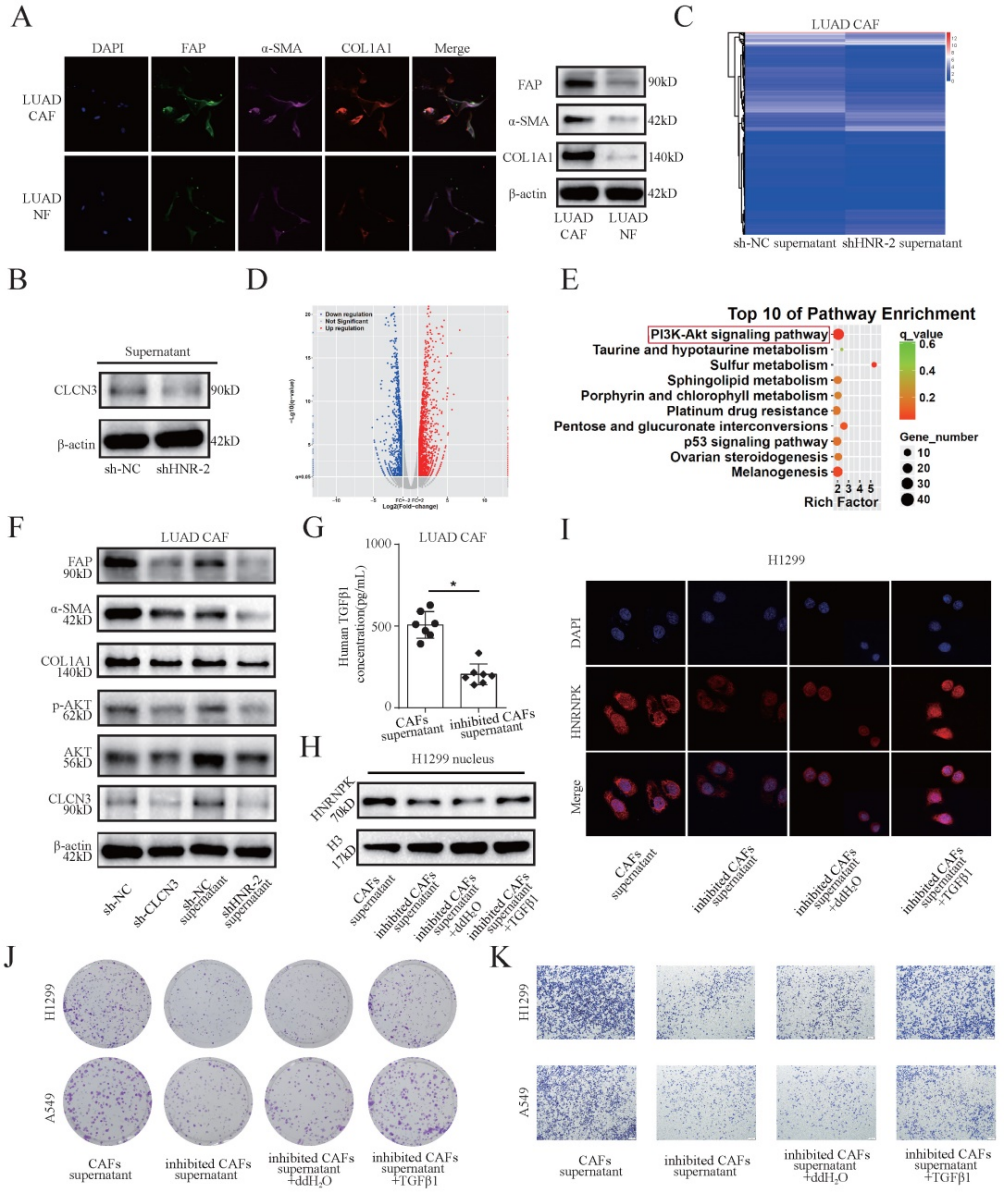
** HNRNPK/CLCN3 axis facilitated LUAD progression through interaction between tumor cells and CAFs. (a)** Primary human CAFs and paired NFs were extracted from fresh LUAD samples. The expression of CAF markers was increased in CAFs compared to NFs, which was in accord with the typical characteristic of CAFs. **(b)** The levels of CLCN3 were detected in the culture supernatants of LUAD cells, and the data indicated that decreased extracellular CLCN3 secretion could be induced by HNRNPK knockdown. **(c-e)** The supernatants (HNRNPK knockdown and control) of LUAD cells were incubated with CAFs for 24 h. We confirmed that the PI3K-AKT signaling pathway was significantly enriched in CAFs. **(f)** The levels of p-AKT, α-SMA, FAP, and COL1A1 were decreased when CAFs were stimulated with the supernatants of HNRNPK-knockdown cells. After CLCN3 knockdown in CAFs, the levels of p-AKT, α-SMA, FAP, and COL1A1 were also effectively inhibited. **(g)** Due to the activation inhibition, the CAFs co-cultured with HNRNPK-knockdown LUAD cells were then considered as inhibited CAFs. The analysis by ELISA revealed that the TGF-β1 production of inhibited CAFs was significantly decreased (*n* = 7). **(h)** The supernatants of inhibited CAFs attenuated the expression of HNRNPK protein in the H1299 nucleus, and the attenuation effect was reversed after TGF-β1 treatment. **(i)** The supernatants of inhibited CAFs attenuated the fluorescence intensity of HNRNPK in the H1299 nucleus, which was also reversed after the addition of exogenous TGF-β1. **(j, k)** The supernatants of inhibited CAFs markedly attenuated the clonogenicity and invasion of LUAD cells, and this phenomenon was further reversed after the addition of TGF-β1 (*n* = 3). *P < 0.05.

Cancer-associated fibroblasts (CAFs) are the predominant components in the stroma of LUAD. To examine the relationship between CLCN3 and the tumor microenvironment, primary human CAFs and paired NFs were isolated from fresh LUAD samples. First, the expression of CAF markers (α-SMA, FAP, and COL1A1) in CAFs and NFs was detected. WB and IF results revealed that the α-SMA, FAP, and COL1A1 expression level was elevated in CAFs in contrast with that in NFs, which was consistent with the typical characteristics of CAFs (**Fig. 4a**). Subsequently, we detected the levels of CLCN3 in the culture supernatants of LUAD cells, and the data indicated that decreased extracellular CLCN3 secretion could be induced by HNRNPK knockdown (**Fig. 4b**). Furthermore, when CAFs were incubated in the supernatants (HNRNPK knockdown and control) of LUAD cells for 24 h, KEGG analysis of CAFs confirmed that the PI3K-AKT signaling pathway was significantly enriched after stimulation of LUAD supernatants (**Fig. 4c, 4d, 4e**). Therefore, we speculated that the secreted CLCN3 could modulate the function of CAFs through the PI3K-AKT signaling pathway. Subsequently, the CAFs were co-cultured with the conditioned medium of HNRNPK-knockdown LUAD cells for 24 h. WB illustrated that the levels of α-SMA, FAP, p-AKT, and COL1A1 were significantly decreased when CAFs were stimulated with the supernatants of HNRNPK-knockdown cells. After the shRNA-mediated knockdown of CLCN3 in CAFs, the levels of p-AKT, α-SMA, FAP, and COL1A1 were also effectively inhibited, which revealed that decreased levels of endogenous or extracellular secreted CLCN3 inhibited CAFs activation through the PI3K-AKT signaling pathway (**Fig. 4f**).

Due to the inhibition of activation, the CAFs co-cultured with HNRNPK-knockdown LUAD cells were considered as inhibited CAFs and the supernatants were collected for further experiments. The analysis by the ELISA kit revealed that the TGF-β1 production of inhibited CAFs was significantly decreased (**Fig. 4g**). Furthermore, by extraction of nucleoprotein, we found that the supernatants of inhibited CAFs attenuated the expression of HNRNPK protein in H1299 nucleus, and the attenuation effect was reversed after TGF-β1 treatment for 6 h (**Fig. 4h**). IF staining suggested that the supernatants of inhibited CAFs attenuated the fluorescence intensity of HNRNPK in H1299 nucleus, which was also reversed after the addition of exogenous TGF-β1 (**Fig. 4i**). Importantly, the supernatants of inhibited CAFs markedly ameliorated the clonogenicity, invasion, and migration of LUAD cells, and this phenomenon was reversed after the addition of TGF-β1. These findings validated that the LUAD progression correlated with intercellular interactions (**Fig. 4j, 4k, S5a-S5e**). Therefore, our data support that the HNRNPK/CLCN3 axis facilitated LUAD progression through the interaction between CAFs and tumor cells.

### HNRNPK regulated the expression and function of CLCN3 *in vivo*

**Figure 5:**
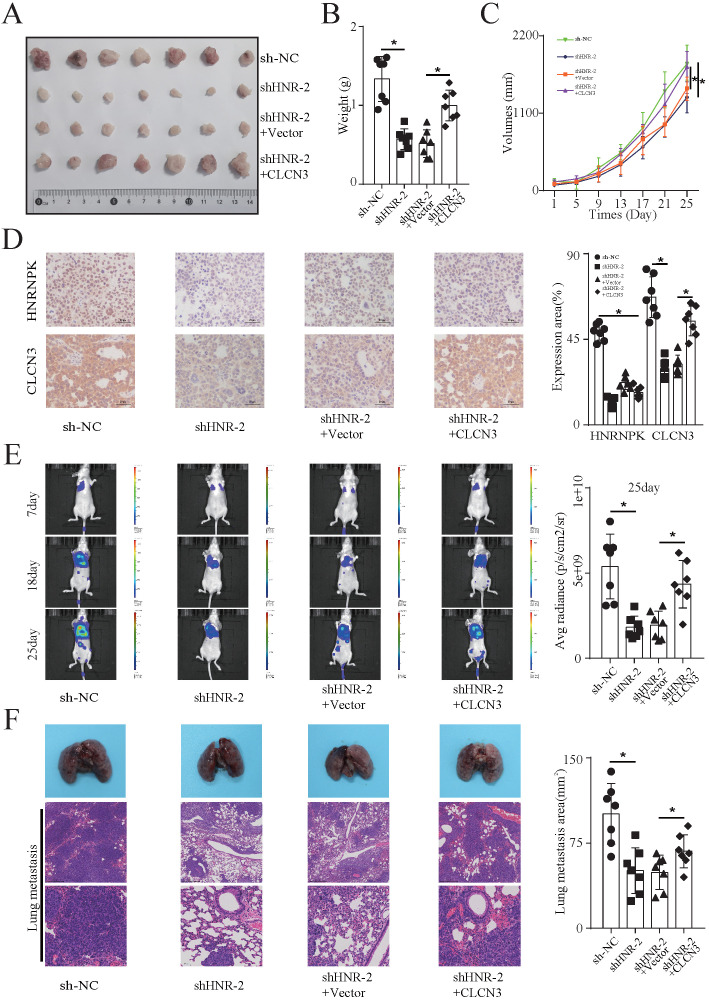
** HNRNPK regulated the expression and function of CLCN3 *in vivo*. (a)** In mouse xenograft models, stable HNRNPK-knockdown cells (H1299) and rescue model cells were administered to the nude mice via subcutaneous injection into their left flank. The representative figure of tumors formed in each group is shown (*n* = 7). **(b, c)** The tumor weight and tumor growth curve were suppressed when HNRNPK was knocked down, and this inhibition was reversed by CLCN3 overexpression (*n* = 7). **(d)** In tumor xenografts, the expression of CLCN3 was decreased following HNRNPK knockdown, however, this reduction was reversed by CLCN3 overexpression (*n* = 7). **(e)** Nude mice received an injection with stable HNRNPK-knockdown cells (H1299) and rescue model cells into their tail veins. HNRNPK knockdown effectively decreased the average radiance of lung metastatic lesions, and the reduction was rescued by CLCN3 overexpression (*n* = 7). **(f)** In paraffin sections of nude mouse lung metastases, we found that HNRNPK knockdown caused a reduction of lung metastasis area, which was rescued by CLCN3 overexpression (*n* = 7). *P < 0.05.

The functional regulation of CLCN3 was then explored *in vivo*. In the H1299 cell line, the promoter activity and regulatory mechanism of CLCN3 were thoroughly investigated. Hence, we chose H1299 cells to establish xenograft-bearing mouse models. Nude mice received a subcutaneous injection of stable HNRNPK-knockdown cells as well as rescue model cells into their left flank. Every four days, measurements were obtained to record the volume of the tumor. Following a period of 25 days, the tumor xenografts were removed and subjected to further processing. The representative figure of tumors formed in each group is shown in Figure 5A (**Fig. 5a**). We observed that the tumor weight and growth were significantly suppressed following HNRNPK knockdown, and the inhibitory influence was abrogated by CLCN3 overexpression (**Fig. 5b, 5c**). In addition, IHC staining of CLCN3 and HNRNPK in tumor xenografts revealed that the expression of CLCN3 was decreased after HNRNPK silencing but that this reduction could be restored by CLCN3 upregulation (**Fig. 5d**). Subsequently, to observe the functional regulation of CLCN3 on tumor metastasis *in vivo*, stable HNRNPK-knockdown cells (H1299) and rescue model cells were transfected with luciferase plasmids and then injected into nude mice's tail veins. Thereafter, the lung metastatic lesion was assessed using bioluminescence imaging technology after 7, 18, and 25 days. The results showed that HNRNPK knockdown effectively decreased the average radiance of the lung metastatic lesion but that this reduction was abrogated by CLCN3 upregulation (**Fig. 5e, S6b**). After 25 days, we used isolated lung metastases from the experimental nude mice, embedded them in paraffin, sectioned the tumors, and performed H&E staining. Similarly, HNRNPK knockdown caused a reduction of the lung metastasis area, which was also rescued by CLCN3 overexpression (**Fig. 5f, S6a**). Thus, we established that the expression and function of CLCN3 were modulated by HNRNPK *in vivo*.

### CLCN3 and HNRNPK were upregulated in LUAD and correlated with poor prognosis

**Figure 6:**
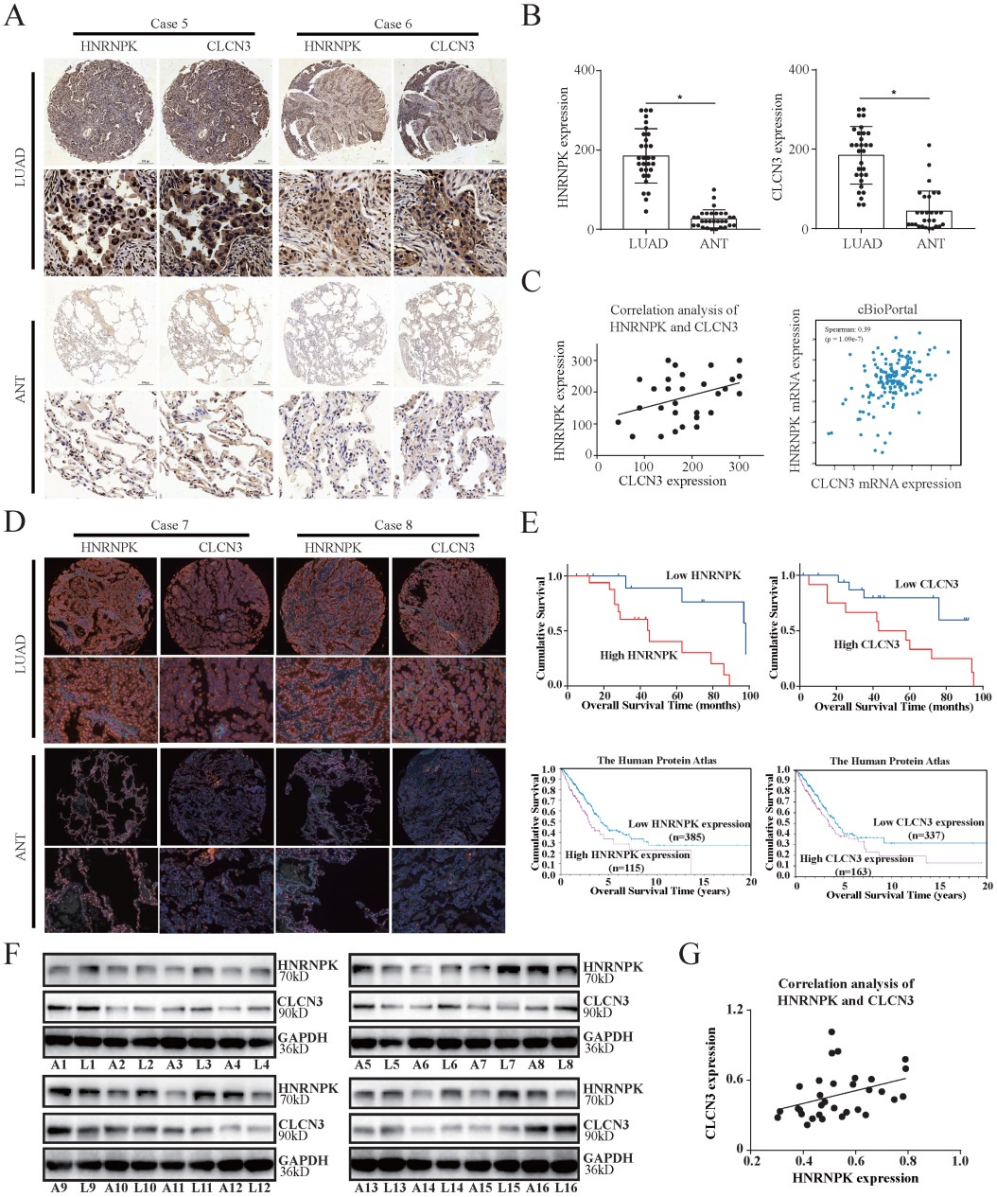
** CLCN3 and HNRNPK were upregulated in LUAD and correlated with poor prognosis. (a, b)** In paraffin-embedded LUAD tissue microarray, we discovered that the expression of CLCN3 and HNRNPK was elevated in LUAD tissues compared with ANT (*n* = 30). **(c)** The CLCN3 expression positively correlated with HNRNPK expression in LUAD tissues. **(d)** Using IF staining of LUAD tissue microarray, we found that both CLCN3 and HNRNPK were highly expressed in LUAD tissues (*n* = 30). **(e)** The Kaplan-Meier survival analysis illustrated that elevated CLCN3 or HNRNPK expression levels in tumors predicted a dismal prognosis for LUAD patients. In the HPA database, the data also suggested that the patients having an elevated expression of CLCN3 or HNRNPK exhibited a shorter overall survival. **(f)** LUAD and adjacent normal tissues were collected (16 cases), and the increased expression of CLCN3 or HNRNPK was confirmed in LUAD tissues (n = 16). **(g)** Correlation analysis indicated a positive expression correlation between CLCN3 and HNRNPK in 16 cases of LUAD tissues. *P < 0.05.

The significant association between CLCN3 and HNRNPK *in vitro* and *in vivo* led us to additionally evaluate their fundamental clinical values. IHC analysis of 30 LUAD tissue samples in a paraffin-embedded microarray was performed to verify the association between CLCN3 and HNRNPK expression. The findings highlighted that the CLCN3 and HNRNPK expression was elevated in LUAD tissues in contrast with ANT (**Fig. 6a, 6b**). Furthermore, the spearman rank correlation analysis illustrated a positive correlation between the CLCN3 and HNRNPK expression in LUAD tissues (*r* = 0.371, *P* < 0.05) (**Fig. 6c, left**). In cBioPortal and GEPIA databases, we also demonstrated that CLCN3 and HNRNPK positively correlated at the RNA level (**Fig. 6c, right, S6c**). Next, the relationship between CLCN3 or HNRNPK and clinicopathological characteristics of LUAD patients was analyzed. We discovered that a correlation existed between higher HNRNPK expression and deeper tumor invasion (*P* = 0.019), and a correlation between greater CLCN3 expression and an elevated probability of lymph node metastases (*P* = 0.027) (**Fig. S6e, S6f**). Moreover, using IF staining of LUAD tissue microarray, we found that both CLCN3 and HNRNPK were highly expressed in LUAD tissues (**Fig. 6d**). Importantly, the Kaplan-Meier survival analysis illustrated that elevated CLCN3 or HNRNPK expression in tumors was linked to poor prognosis for LUAD patients (*P* < 0.05) (**Fig. 6e, upper**). Additionally, the analysis of data from the Human Protein Atlas (HPA) database suggested that the patients having elevated CLCN3 or HNRNPK levels exhibited a shorter OS duration (*P* < 0.05) (**Fig. 6e, below**). Meanwhile, we collected 16 cases of LUAD tissues and ANT, and the increased CLCN3 or HNRNPK expression was confirmed in LUAD tissues (**Fig. 6f, S6d**). Further correlation analysis indicated a positive expression correlation between CLCN3 and HNRNPK in LUAD tissues (*r* = 0.374, *P* < 0.05) (**Fig. 6g**). In total, our results indicated that CLCN3 and HNRNPK were upregulated in LUAD and correlated with poor prognosis.

## Discussion

Membrane chloride channels are often abnormally expressed in several kinds of tumors and have a role in the modulation of a variety of behaviors that are associated with cancer cells. Additionally, chloride channels have the potential to be useful biological markers for cancer 17, 18. One chloride channel family member CLCN3 is an essential exchange transporter located both in the plasma membrane and cytoplasm 6. In the plasma membrane, CLCN3 functions as a chloride channel and regulates cell proliferation and apoptosis. In the cytoplasm, CLCN3 is mainly distributed in late endosomes and lysosomes, where it helps regulate the acidity of intracellular vesicles, which mediate drug resistance. This study focused on total cellular CLCN3 and identified that the primary biological functions associated with CLCN3 were locomotion and growth, which agreed with the clinicopathological analysis of CLCN3 expression in LUAD patients. For this reason, we speculate that through its ability to enhance both cell proliferation and migration, CLCN3 in LUAD may be considered a possible factor that encourages the development of tumors. Although many signal transduction molecules and networks were found in the KEGG pathway analysis of CLCN3, the main signaling pathway affecting proliferation and migration was not screened yet. The identification of the related signal pathway needed more research.

HBE, a normal cell line isolated from human bronchial surface epithelium, is usually used as the normal control to study the pathology of the respiratory system as well as lung cancer 19-21. In this investigation, when LUAD cells and tissues were contrasted with normal cells and tissues, we discovered that CLCN3 levels were much higher in the LUAD. It was unknown, however, which core transcription factor regulated CLCN3 expression in LUAD. Given that regulatory processes frequently take place at gene promoters 22, we hypothesized that a specific transcriptional regulation mechanism for CLCN3 might exist in LUAD. Moreover, some transcription factors found in tumors might bind to the CLCN3 promoter to enhance CLCN3 expression. Hence, a DNA probe containing the CLCN3 promoter region was generated to identify promoter-binding transcription factors. We observed that many differentially expressed proteins were pulled down. Because the band with the lower molecular weight was more likely to seek out and identify transcription factors, we began our investigation by focusing on the 75 kDa band rather than the 100 kDa or 130 kDa band. Our data eventually demonstrated that transcription factor HNRNPK bound the CLCN3 promoter 23. The discovery that HNRNPK is a protein that binds to the CLCN3 promoter was the most important and unique finding in this study.

To verify the interaction between the CLCN3 promoter and HNRNPK, a ChIP assay was carried out to investigate the fragment of the CLCN3 promoter that HNRNPK bound. The ChIP-seq data showed that the recruitment of HNRNPK was mostly enriched at the CLCN3 promoter region (-538/+226bp). Moreover, HNRNPK knockdown markedly suppressed the promoter activity of the pGL4.10-CLCN3-538 reporter plasmid, while the promoter activities of the pGL4.10-CLCN3-822 and pGL4.10-CLCN3-248 plasmids were not significantly decreased, indicating that the possible binding position was likely positioned between -248 and -538. The binding motif 'GCGAGG' from ChIP-seq data was indeed enriched in this region. Furthermore, HNRNPK knockdown decreased the CLCN3 RNA level, demonstrating that HNRNPK was responsible for modulating the expression of CLCN3 at the transcriptional level.

HNRNPK, a member of the hnRNP family, participates in diverse key cellular activities, such as nuclear non-coding RNA regulation, carcinogenesis, and bone formation 13. In addition to this, it is a protein that binds conserved pre-mRNA and is implicated in many different phases of gene expression, such as genome stability, mRNA splicing, chromatin remodeling, transcription, and translation 24-26. Numerous research reports have demonstrated HNRNPK as an oncogene that is closely linked to the prognosis of many malignant tumors 27. Recent research has illustrated that HNRNPK acts as a tumor suppressor in both hematological and gastric cancers 28, 29. Thus, HNRNPK has the potential to serve dual functions, either preventing or enhancing tumor growth, depending on the kind of tumor it is acting on. In LUAD, the specific functions of HNRNPK remain incompletely understood. Here, we discovered that there was an increase in the expression of HNRNPK in LUAD, which was in line with the changes seen in CLCN3. Furthermore, consistent with the results of Li et al. 30, patients who exhibited elevated levels of HNRNPK had unfavorable prognoses. The clinicopathological characteristics suggested that HNRNPK may enhance the proliferative and invasive ability of LUAD cells. Given that the function of HNRNPK is similar to that of CLCN3 and that H1299 and A549 cells have a relatively higher basal expression of CLCN3 than PC-9 cells, we performed HNRNPK-knockdown experiments in H1299 and A549 cells, and HNRNPK-overexpression experiments in PC-9 cells. The key biological activities of CLCN3, which were reduced following HNRNPK downregulation and elevated following HNRNPK upregulation *in vitro*, demonstrate that HNRNPK performs a role in the promotion of tumor growth*.* Both the rescue models in which CLCN3 was upregulated and the reverse models in which CLCN3 was downregulated provided additional confirmation that CLCN3 was the target of HNRNPK. Additionally, we examined the relationship between CLCN3 and HNRNPK in mouse xenograft models, which also showed that the function and expression regulation of CLCN3 were regulated by HNRNPK *in vivo*. The reasonable inference drawn from all of these findings is that HNRNPK is responsible for modulating both the expression and the function of CLCN3. However, LUAD cells might contain other molecular regulators that remain to be identified.

During tumorigenesis, the TME undergoes considerable alterations in both its composition and its physical characteristics, and the secretome of the cancer cells performs crucial functions in this process 31. Previous studies have indicated that the chloride intracellular channel protein 3 (CLIC3), which is a protein that is released by cancer cells and may be identified in high numbers in both the stromal and tumor components of malignant ovarian tumors 32. CLIC3 proteins are mostly found inside the cells because they lack the membrane-spanning domains that are typical of channel proteins 33. It could be speculated that the CLCN3, a voltage-gated chloride transmembrane protein, may be more likely to be secreted into the TME. However, there are no studies on the relationship between CLCN3 and TME. In this work, we first found secreted CLCN3 in LUAD supernatants. Since the CLCN3 protein was mainly distributed in late endosomes, lysosomes, and the plasma membrane, we speculated that CLCN3 might be secreted because of multivesicular endosomes' fusion with the cell surface. The PI3K/AKT signaling pathway is fundamental to cell differentiation, growth, apoptosis, and mobility 34. Abundant data suggests that the PI3K/AKT pathway induces the transition of diverse cells into CAFs and exhibits a role in promoting CAFs activation 35. After CLCN3 modulated the activation of CAFs, the findings of both the western blot and the RNA-seq experiment indicated that the PI3K-AKT signaling pathway was considerably altered. Important upstream modulators of the PI3K-AKT signaling pathway are proteins called receptor tyrosine kinases (RTKs) 36. The fibroblast growth factor receptor (FGFR) family is a member of receptor tyrosine kinases (RTKs) and has been critical in CAFs activation 37. Therefore, we speculated that secreted CLCN3 might bind to the FGFR and result in CAF activation through the PI3K-AKT signaling pathway. The FGFR family is composed of four receptor tyrosine kinases. Thus, future studies will be focused on exploring and validating potential specific binding molecules.

CAFs are prevalent in the stroma and exert a crucial function in the formation of a TME that is abnormal and permissive to the onset and advancement of carcinomas 38. Commonly used markers to define CAFs are αSMA and FAP 15. Additionally, COL1A1, S100A4, vimentin, and PDGFRβ can also serve as markers for CAFs 39, 40. However, these markers are nonspecific, indicating that more combinations of them might be useful to identify CAFs. In this study, two typical markers (αSMA and FAP) and COL1A1 were chosen to identify CAFs. It has been reported that the production of soluble factors particularly SDF1/CXCL12 and TGFβ from CAFs can drive cancer progression and the activation of CAFs is linked to the transforming growth factor-beta 1 (TGF-β1) levels 41, 42. Therefore, we attempted to measure the TGF-β1 production by CAFs. Our data demonstrated that decreased extracellular CLCN3 secretion inhibited CAFs activation and TGF-β1 production. However, we cannot exclude the possibility that other cytokines or proteins might also mediate the intercellular communication in the TME. A comprehensive analysis and large-scale screen of the factors are required in the future.

In colorectal and prostate cancers, HNRNPK in the nucleus enhance the transcriptional activity of MMP2 to facilitate cell invasion 43. In pancreatic cancer, the nuclear localization signal of HNRNPK transcriptionally drives the activation of YAP1 and regulates cancer progression 44. In NSCLC, the nuclear HNRNPK can promote the epithelial mesenchymal transition of cancer cells 30. On this basis, we speculated that CAFs might mediate the nuclear signal of HNRNPK in LUAD cells. Our data revealed that the supernatants of inhibited CAFs attenuated the expression and fluorescence intensity of HNRNPK in the nucleus, thus suppressing the progression of LUAD. Furthermore, this phenomenon was rescued and reversed after the addition of TGF-β1, which validated that the CLCN3-TGFβ1-HNRNPK axis facilitated LUAD progression through interaction between tumor cells and CAFs. Accumulating evidence has revealed that the crosstalk between CAFs and tumor cells exists in multiple cancer types. By providing a growth niche, secreting cytokines, inducing chemoresistance, or promoting immune evasion, CAFs communicate with tumor cells and perform integral functions in boosting tumor progression. For example, in gastric cancer, the interaction between activated fibroblasts and tumor cells mediated by IL-33 signaling promoted tumor metastasis 45. In bladder cancer, the CXCL1-mediated interaction of tumor cells with CAFs promoted tumor growth 46. Overall, our findings suggest that the HNRNPK/CLCN3 axis might act as an important mediator of CAF-tumor interaction in LUAD progression.

**Figure 7:**
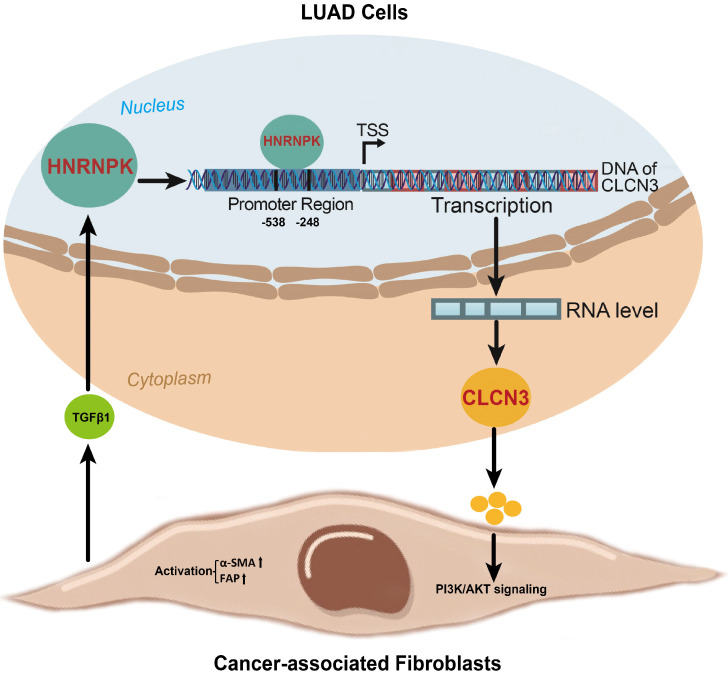
** A schematic representation of the relationship between CLCN3 and HNRNPK in LUAD progression.** Firstly, CLCN3 was upregulated in human LUAD and facilitated tumor proliferation and migration. Secondly, HNRNPK was validated as a CLCN3 promoter-binding transcription factor, and the binding motif 'GCGAGG' and binding site '-538/-248 bp' were identified. Finally, the expression and function of CLCN3 were regulated by HNRNPK *in vitro* and *in vivo*, and the HNRNPK-CLCN3 axis facilitated LUAD progression in a feedback way through CAF-tumor interaction in the TME.

In this work, the focus was to determine the changes in expression of the HNRNPK/CLCN3 axis in LUAD cells. In view that the primary function and prognostic role of HNRNPK were the same as CLCN3, we considered that both HNRNPK and CLCN3 perform equally significant functions in LUAD progression, and the therapeutic potential of targeting HNRNPK and targeting CLCN3 might be equal. Here, we provide a schematic representation of the relationship between CLCN3 and HNRNPK in LUAD (**Fig. 7**). Firstly, CLCN3 was upregulated in human LUAD and facilitated tumor proliferation and migration. Secondly, HNRNPK was validated as a CLCN3 promoter-binding transcription factor, and the binding motif 'GCGAGG' and binding site '-538/-248 bp' were identified. Finally, the expression and function of CLCN3 were regulated by HNRNPK *in vitro* and *in vivo*, and the HNRNPK-CLCN3 axis facilitated LUAD progression in a feedback way through CAF-tumor interaction in the TME.

## Conclusions

HNRNPK/CLCN3 axis facilitates the progression of LUAD through CAF-tumor interaction. This study uncovers novel molecular biological mechanisms of LUAD progression and may provide novel therapeutic targets.

## Supplementary Material

Supplementary figures.

## Abbreviations

LUAD: lung adenocarcinoma
CLCN3: chloride channel 3
HNRNPK: heterogeneous nuclear ribonucleoprotein K
CAFs: cancer associated fibroblasts
TSS: transcription start sites
GEO: gene expression omnibus
GO: gene ontology
KEGG: kyoto encyclopedia of genes and genomes
NSP: nonspecific probe
ChIP: chromatin immunoprecipitation
MS: mass spectrometry
IP: immunoprecipitation
WB: western blot
IHC: immunohistochemistry
IF: immunofluorescence
ANT: adjacent normal tissues
