# Supplementary material for: HNRNPK/CLCN3 axis facilitates the progression of LUAD through CAF-tumor interaction
Source: Int J Biol Sci. 2022 Oct 17;18(16):6084–101. doi: 10.7150/ijbs.76083 (PMC9682536; doi:10.7150/ijbs.76083)
Supplement: Supplementary file 1 — Supplementary figures. [file ijbsv18p6084s1.pdf]

Supplementary Figure Legends

Figure S1. CLCN3 was upregulated in human LUAD and facilitated tumor proliferation and migration. (a) The basic protein expression of CLCN3 was detected in human LUAD cell lines and human bronchial epithelial cell lines. **(b)** The protein expression of CLCN3 was suppressed when CLCN3 was knocked down in H1299 and A549 cells (n = 3). **(c)** The heatmap plot and KEGG pathway analysis of RNA-seq were constructed after CLCN3 knockdown in H1299 cells. **(d)** The representative cell images showed that CLCN3 knockdown inhibited cell proliferation at 24 h. **(e)** Knockdown of CLCN3 suppressed the proliferative ability of A549 and H1299 cells at 24 h (n = 3). **(f)** Knockdown of CLCN3 inhibited the migration of A549 and H1299 cells (n = 3). *$P < 0.05$

Figure S2. HNRNPK was identified and validated as a CLCN3 promoter-binding transcription factor. (a) The fragmented DNA was extracted, and the ChIP-seq DNA libraries were successfully constructed. The standard pie chart of peak annotation illustrated that over half of the peaks were located within the enhancer regions (intronic and intergenic regions) whereas 6.71% of the peaks were located in the promoter regions. **(b)** The typical transcription start site (TSS) enrichment plot showed that the IP fragments are mainly enriched at TSS, compared to the input fragments. **(c)** The ChIP-qPCR data showed that the binding of HNRNPK to the CLCN3 gene promoter (-538/-248 bp) was significantly increased in the IP group (n = 3). **(d, e)** GSEA demonstrated that the growth and locomotory behavior significantly correlated with HNRNPK knockdown. **$P < 0.01$

Figure S3. HNRNPK knockdown inhibited the expression and function of CLCN3 *in vitro*. (a, b) To precisely monitor cell proliferation, an analysis based on IncuCyte was conducted. The analysis of the results showed that the level of cell proliferation was reduced following HNRNPK knockdown and that this reduction could be restored by increasing CLCN3 expression levels. **(c, d)** The cell migration was suppressed after knocking down HNRNPK, however, this inhibition was abrogated by CLCN3 overexpression (n = 3). **(e)** IncuCyte data showed that the proliferation of LUAD cells was promoted following HNRNPK overexpression but this effect was attenuated by CLCN3 knockdown. *$P < 0.05$

Figure S4. HNRNPK overexpression promoted the expression and function of CLCN3 *in vitro*. (a) After HNRNPK was overexpressed, there was an elevation in the expression level of CLCN3, and knocking down CLCN3 levels had the effect of reversing the elevation. **(b, c)** Following HNRNPK overexpression, LUAD cells exhibited enhanced levels of clonogenicity and proliferation, but these enhanced levels were abrogated by CLCN3 knockdown. (n = 3). **(d, e)** Cell invasion and migration were enhanced following HNRNPK overexpression, however, the consequences of this enhancement were reversed when CLCN3 was knocked down (n = 3). *$P < 0.05$

Figure S5. HNRNPK/CLCN3 axis facilitated LUAD progression through interaction

between tumor cells and CAFs. (a, b) The supernatants of inhibited CAFs markedly attenuated the cell clonogenicity of LUAD, and this phenomenon was rescued after the addition of TGF-β1 ($n = 3$). **(c)** The supernatants of inhibited CAFs markedly attenuated the cell invasion of LUAD, and this phenomenon was rescued by the addition of TGF-β1 ($n = 3$). **(d, e)** The supernatants of inhibited CAFs markedly attenuated the cell migration of LUAD, and this phenomenon was rescued after the addition of TGF-β1 ($n = 3$). $^*P < 0.05$

Figure S6. HNRNPK regulated CLCN3 *in vivo*, and both were upregulated in human LUAD. (a) In paraffin sections of nude mouse lung metastases, we found that HNRNPK knockdown caused a reduction of lung metastasis area, which was rescued by CLCN3 overexpression ($n = 7$). **(b)** HNRNPK knockdown effectively decreased the average radiance of lung metastatic lesions, and the reduction was abrogated by CLCN3 overexpression ($n = 7$). **(c)** In the GEPIA database, CLCN3 and HNRNPK positively correlated at the RNA level. **(d)** LUAD tissues and adjacent normal tissues were collected (16 cases), and the increased expression of CLCN3 or HNRNPK was confirmed in LUAD tissues ($n = 16$). **(e, f)** The relationship between CLCN3 or HNRNPK and clinicopathological characteristics of LUAD patients was analyzed. We found that higher HNRNPK expression correlated with deeper tumor invasion, and higher CLCN3 expression correlated with enhanced lymph node metastases. $^*P < 0.05$

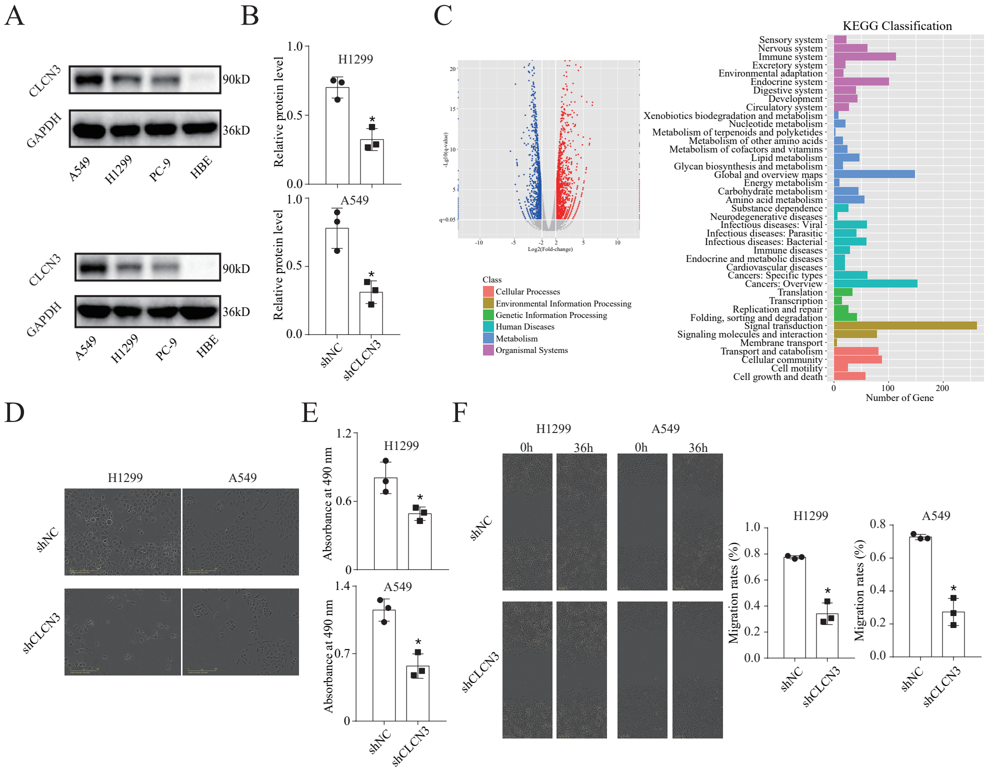
A
B
C
D
E
F
CLCN3
GAPDH
90kD
36kD
A549
H1299
PC-9
HBE
Relative protein level
shNC
shCLCN3
KEGG Classification
Sensory system
Nervous system
Immune system
Excretory system
Environmental adaptation
Endocrine system
Digestive system
Development
Circulatory system
Xenobiotics biodegradation and metabolism
Nucleotide metabolism
Metabolism of terpenoids and polyketides
Metabolism of other amino acids
Metabolism of cofactors and vitamins
Lipid metabolism
Glycan biosynthesis and metabolism
Global and overview maps
Energy metabolism
Carbohydrate metabolism
Amino acid metabolism
Substance dependence
Neurodegenerative diseases
Infectious diseases: Viral
Infectious diseases: Parasitic
Infectious diseases: Bacterial
Immune diseases
Endocrine and metabolic diseases
Cardiovascular diseases
Cancers: Specific types
Cancers: Overview
Translation
Transcription
Replication and repair
Folding, sorting and degradation
Signal transduction
Signaling molecules and interaction
Membrane transport
Transport and catabolism
Cellular community
Cell motility
Cell growth and death
Number of Gene
-Lg10(q-value)
Log2(Fold-change)
q=0.05
Class
Cellular Processes
Environmental Information Processing
Genetic Information Processing
Human Diseases
Metabolism
Organismal Systems
Absorbance at 490 nm
0h
36h
Migration rates (%)

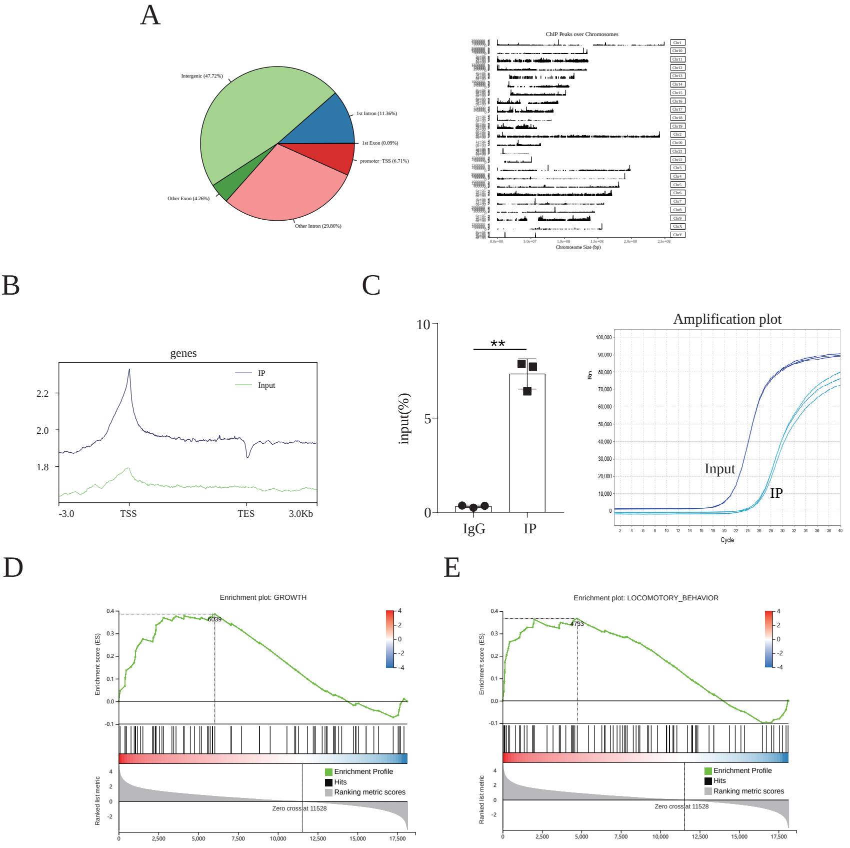
A
ChIP Peaks over Chromosomes
Intergenic (47.72%)
1st Intron (11.36%)
1st Exon (0.09%)
promoter−TSS (6.71%)
Other Exon (4.26%)
Other Intron (29.86%)
Chromosome Size (bp)
B
genes
IP
Input
2.2
2.0
1.8
-3.0
TSS
TES
3.0Kb
C
10
5
0
input(%)
**
IgG
IP
Amplification plot
Input
IP
Rn
Cycle
D
Enrichment plot: GROWTH
Enrichment score (ES)
6039
Enrichment Profile
Hits
Ranking metric scores
Ranked list metric
Zero cross at 11528
E
Enrichment plot: LOCOMOTORY_BEHAVIOR
Enrichment score (ES)
4733
Enrichment Profile
Hits
Ranking metric scores
Ranked list metric
Zero cross at 11528

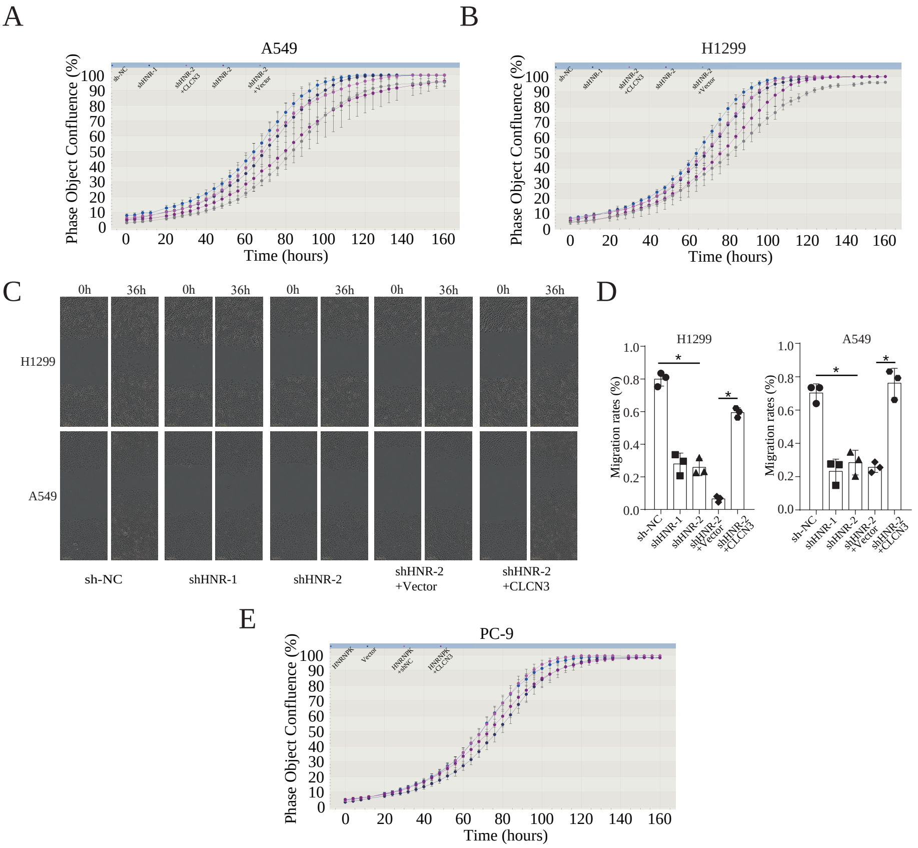
A
A549
Phase Object Confluence (%)
Time (hours)
sh-NC
shHNR-1
shHNR-2 +CLCN3
shHNR-2
shHNR-2 +Vector
B
H1299
Phase Object Confluence (%)
Time (hours)
C
0h
36h
H1299
A549
sh-NC
shHNR-1
shHNR-2
shHNR-2 +Vector
shHNR-2 +CLCN3
D
H1299
A549
Migration rates (%)
sh-NC
shHNR-1
shHNR-2
shHNR-2 +Vector
shHNR-2 +CLCN3
E
PC-9
Phase Object Confluence (%)
Time (hours)
HNRNPK
Vector
HNRNPK +shNC
HNRNPK +CLCN3

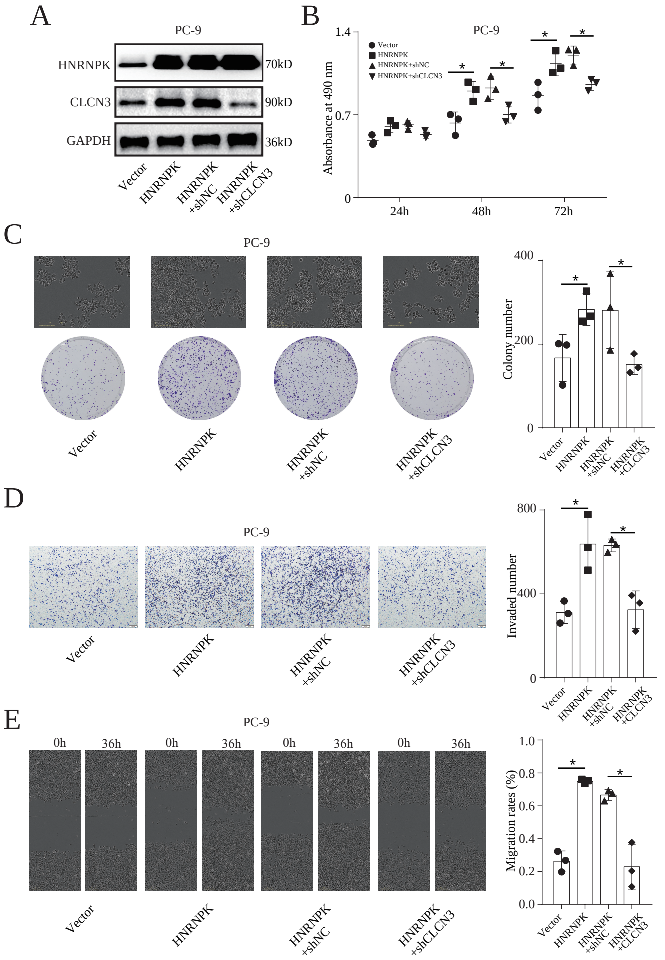
A
PC-9
HNRNPK
CLCN3
GAPDH
70kD
90kD
36kD
Vector
HNRNPK
HNRNPK +shNC
HNRNPK +shCLCN3
B
PC-9
Vector
HNRNPK
HNRNPK+shNC
HNRNPK+shCLCN3
Absorbance at 490 nm
1.4
0.7
0
24h
48h
72h
*
C
PC-9
Vector
HNRNPK
HNRNPK +shNC
HNRNPK +shCLCN3
Colony number
400
200
0
Vector
HNRNPK
HNRNPK +shNC
HNRNPK +CLCN3
D
PC-9
Vector
HNRNPK
HNRNPK +shNC
HNRNPK +shCLCN3
Invaded number
800
400
0
Vector
HNRNPK
HNRNPK +shNC
HNRNPK +CLCN3
E
PC-9
0h
36h
Vector
HNRNPK
HNRNPK +shNC
HNRNPK +shCLCN3
Migration rates (%)
1.0
0.8
0.6
0.4
0.2
0.0
Vector
HNRNPK
HNRNPK +shNC
HNRNPK +CLCN3

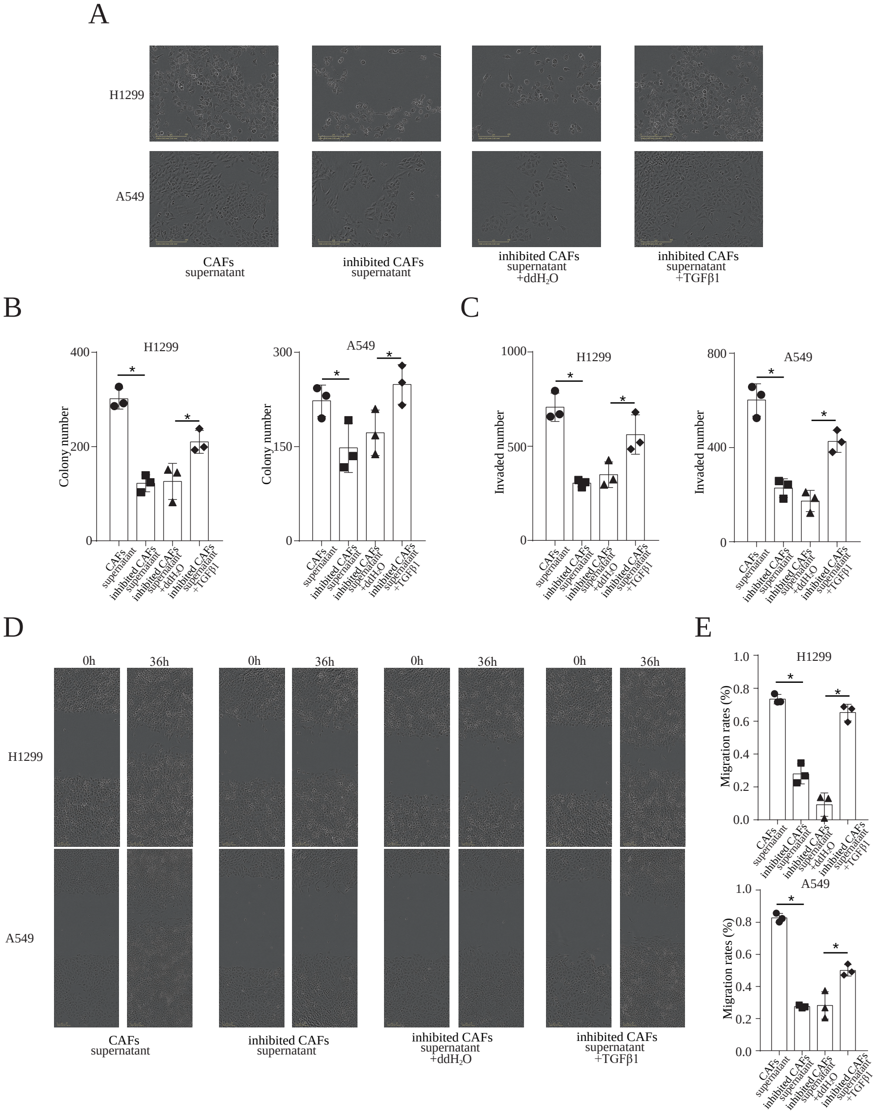
A
H1299
A549
CAFs supernatant
inhibited CAFs supernatant
inhibited CAFs supernatant +ddH2O
inhibited CAFs supernatant +TGFβ1
B
C
H1299
A549
Colony number
Invaded number
D
E
0h
36h
Migration rates (%)

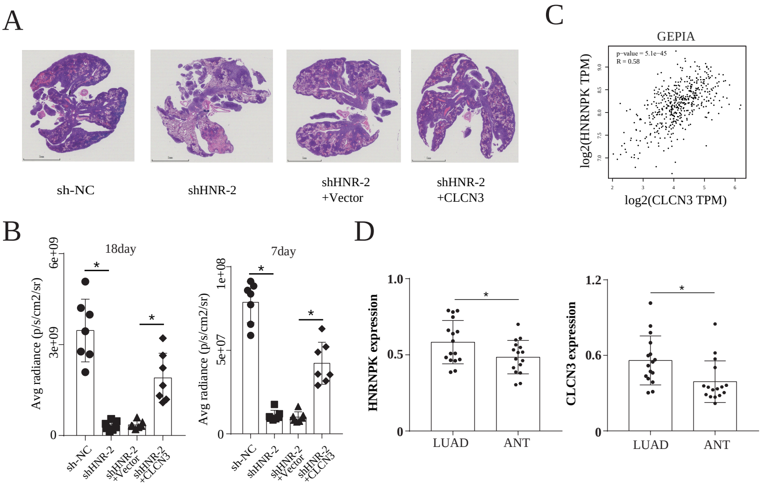

E

Variables	N	HNRNPK low expression	HNRNPK high expression	*P* value
Depth of invasion				
T1/T2	10	8	2	**0.019***
T3/T4	20	6	14	
Lymph node metastases				
N0	8	5	3	0.427
N1/N2/N3	22	9	13	
Distant metastasis				
M0	19	8	11	0.707
M1	11	6	5	
TNM stage				
I/II	14	9	5	0.141
III/IV	16	5	11	

F

Variables	N	CLCN3 low expression	CLCN3 high expression	*P* value
Depth of invasion				
T1/T2	10	3	7	0.694
T3/T4	20	9	11	
Lymph node metastases				
N0	8	6	2	**0.027***
N1/N2/N3	22	6	16	
Distant metastasis				
M0	19	7	12	0.712
M1	11	5	6	
TNM stage				
I/II	14	5	9	0.722
III/IV	16	7	9	
